# Perceptions of Health Care Providers Regarding a Mobile Health Intervention to Manage Chronic Obstructive Pulmonary Disease: Qualitative Study

**DOI:** 10.2196/13950

**Published:** 2019-06-10

**Authors:** Meshari F Alwashmi, Beverly Fitzpatrick, Erin Davis, John-Michael Gamble, Jamie Farrell, John Hawboldt

**Affiliations:** 1 Memorial University of Newfoundland St John's, NL Canada; 2 School of Pharmacy Faculty of Science University of Waterloo Waterloo, ON Canada

**Keywords:** mHealth, COPD, qualitative, smartphone, eHealth, technology

## Abstract

**Background:**

Using a mobile health (mHealth) intervention, consisting of a smartphone and compatible medical device, has the potential to enhance chronic obstructive pulmonary disease (COPD) treatment outcomes while mitigating health care costs.

**Objective:**

The aim of this study was to explore the potential facilitators and barriers among health care providers (HCPs) regarding the use of mHealth interventions for COPD management.

**Methods:**

This was a qualitative study. Semistructured individual interviews were conducted with HCPs, including nurses, pharmacists, and physicians who work directly with patients with COPD. A flexible prompts guide was used to facilitate discussions. Interview topics included the following: demographics, mHealth usage, perceptions toward challenges of mHealth adoption, factors facilitating mHealth adoption, and preferences regarding features of the mHealth intervention for COPD management. Interviews were conversational in nature, and items were not asked verbatim or in the order presented. The interviews were transcribed verbatim and compared against the digital recordings to ensure the accuracy of the content. After creating a codebook for analysis, 2 researchers independently coded the remaining interview data using pattern coding. They discussed commonalities and differences in coding until a consensus was reached.

**Results:**

A total of 30 nurses, physicians, and pharmacists participated. The main facilitators to mHealth adoption are possible health benefits for patients, ease of use, educating patients and their HCPs, credibility, and reducing cost to the health care system. Alternatively, the barriers to adoption are technical issues, privacy and confidentiality issues, lack of awareness, potential limited uptake from the elderly, potential limited connection between patients and HCPs, and finances.

**Conclusions:**

It is important to understand the perceptions of HCPs regarding the adoption of innovative mHealth interventions for COPD management. This study identifies some potential facilitators and barriers that may inform the successful development and implementation of mHealth interventions for COPD management.

## Introduction

### Background

The surge in computing power and mobile connectivity has led to the emergence of mobile health (mHealth) that can transform the mode and quality of clinical research and health care [1]. mHealth is defined by the National Institutes of Health as the use of mobile and wireless devices to improve health outcomes, health care services, and health research. An mHealth intervention could also include the use of a medical device that is compatible with a smartphone. Evidence suggests that mHealth interventions may benefit patients with many chronic health conditions including chronic obstructive pulmonary disease (COPD) [2-5].

Although COPD is a preventable and treatable condition, it is estimated to be the third leading cause of death worldwide by 2020 [6]. According to the Canadian Institute for Health Information, COPD now accounts for the highest rate of hospital admission and readmission among major chronic illnesses in Canada [7]. The Conference Board of Canada has stated that the combined direct and indirect costs of COPD will increase from just under CAD $4 billion in 2010 to roughly $9.5 billion by 2030, an increase of 140% [8]. Dynamic modeling has shown that any intervention that can reduce the number of exacerbations in a population will have a substantial impact on morbidity and costs associated with COPD [8,9]. The authors previously published a systematic review and noted that the current literature on the role of smartphones in reducing COPD exacerbations is limited but does suggest that smartphone interventions may reduce COPD exacerbations [2].

### Importance of Human-Centered Design

The International Organization for Standardization (ISO) 9241-210 standard defines human-centered design (HCD) as “an approach to systems design and development that aims to make interactive systems more usable by focusing on the use of the system and applying human factors/ergonomics and usability knowledge and techniques” [10]. The ISO uses the term HCD instead of user-centered design to “address impacts on a number of stakeholders, not just those typically considered as users” [10]. However, in practice, these terms are often used synonymously.

There is increasing interest from academics and clinicians in harnessing smartphone apps as a means of delivering behavioral interventions for health; however, research on the development and evaluation of such apps is in the relatively early stages [11]. Many of the barriers to using mHealth can be avoided with better planning and collaboration [12]. Testing mHealth interventions with patients has revealed preferences and concerns unique to the tested population [13-15]. When developing an mHealth intervention, Hopkins et al [16] encourage including insights from key users to potentially improve the process and the outcome of the intervention.

Triantafyllidis et al used an iterative approach to refine a tablet computer–based home monitoring system for heart failure patients [17]. There was limited uptake of the system owing to usage difficulties and low levels of patient satisfaction. The authors recommended patient-centered approaches for sustainable delivery of remote health monitoring services [17]. Patient-centered care recognizes the complex, subjective, and changing nature of the patient’s health status [18]; in addition, it links multiple episodes of care offered by diverse providers into continuous, integrated care trajectories unique to particular patients [19,20]. Developing a COPD mHealth intervention with insights from health care providers (HCPs) working with patients with COPD will potentially improve the process and outcome of the mHealth intervention.

### Involvement of Health Care Providers

HCD is particularly suited to developing mHealth interventions, which generally involve multiple stakeholders. Bender suggests a collaborative care approach within teams comprising physicians, nurses, pharmacists, and patient advocates to lead to better care and higher patient adherence for complex and comorbid conditions [21]. Also, other researchers recommend the involvement of a multidisciplinary team in mHealth interventions to develop tailored messages [22], address patient medication needs [23], enhance physical activity in patients with COPD [24], and support the management of heart failure [17], diabetes [25], and cancer [26]. Chiang et al [27] stated that few studies have examined the obstacles faced by HCPs when carrying out telehealth interventions. Similar obstacles in mHealth need to be addressed.

Nursing, medicine, and pharmacy are some of the largest health professions in Canada. Nurses promote COPD management by supportive, preventive, therapeutic, palliative, and rehabilitative means to gain or maintain optimal function [28]. Physicians assess the condition of COPD and diagnose, treat, and prevent any disease, disorder, or dysfunction whereas pharmacists play a role in the promotion of health, prevention, and treatment of COPD through monitoring and management of medication therapy [28]. Furthermore, the role of pharmacists has shifted from drug dispensing responsibilities to the provision of direct patient care [29]. By obtaining the perspectives of nurses, physicians, and pharmacists, we hope to understand the facilitators and barriers affecting some of the largest health professions in Canada. Furthermore, it will enable us to understand the differences in requirements for an mHealth intervention.

### Human-Centered Design in Chronic Obstructive Pulmonary Disease

Although mHealth is gaining popularity in recent years, patient and HCP perspectives toward using mHealth for COPD management are relatively unexplored [30]. One study provided insight into the perceptions of COPD patients and their HCPs toward using mHealth for COPD management. They stated that potential barriers to use mHealth include the following: patients avoiding confrontation with the disease, preference for personal contact with an HCP, difficulties with displaying feelings in an application leading to invalid patient measures and lack of trust in advising characteristics of an mHealth intervention, and lack of enthusiasm for mHealth by HCPs [30]. They also recommended including a larger sample of HCPs with more mHealth experience in future studies [30].

To improve the success of mHealth solutions in COPD management, we suggest including HCPs who work with patients with COPD in the development process. Lessons learned will bridge the knowledge gap of barriers and facilitators for mHealth uptake in COPD management. It will also be offered as a guide for research and technology developers working with COPD patients and their HCPs.

## Methods

### Purpose

This study was intended to explore and develop an understanding of potential facilitators and barriers that might influence HCPs using mHealth interventions for COPD management.

### Study Design

We used a descriptive qualitative research design that was grounded in pragmatism [31,32]. Using a qualitative methodology allowed us to achieve an in-depth, contextualized picture of how a diverse sample of HCPs, in this case nurses, pharmacists, and physicians, think and feel about the possibilities and challenges of using mHealth. This has a pragmatic value as mHealth is an emerging option for delivering health care.

### Recruitment and Study Setting

HCPs involved in the treatment of patients with COPD were eligible to participate. The primary investigator (PI) contacted the Newfoundland and Labrador Medical Association, the Association of Registered Nurses of Newfoundland and Labrador, and the Pharmacists' Association of Newfoundland and Labrador. These organizations were asked to forward a recruitment email to their mailing lists or post it in their websites. Interested HCPs contacted the PI via email or telephone, who then scheduled appointments to complete the consent forms and conduct the interviews. Our sample consisted of 30 HCPs: 10 nurses, 10 pharmacists, and 10 physicians. The study took place in St. John’s, Canada. We conducted some interviews at Memorial University and others at the participants’ offices or homes.

We used purposive typical case sampling to gather information that would reflect typical cases of mHealth use [32,33]. We also used a criterion-based selection [32] so that we could categorize participant characteristics such as age, familiarity with mHealth, health care profession, and years of experience. In addition, as the interviews progressed, some participants were recruited by snowball or chain sampling, where participants suggested other possible HCPs [32,34]. Snowball or chain sampling was used to ask a few information-rich participants for additional contacts to provide confirming or different perspectives, allowing for richer data [32].

Participants were recruited from April 2018 to August 2018. We first contacted nurses, and after interviewing 7 to 8 nurses, we reached saturation as we were not gathering new information. However, we continued interviewing until 10 nurses were interviewed. This was to strengthen the validity of inferences [35]. We used the same sampling strategy for the remaining professions, with similar saturation points and continuing to interview the 10 participants for each profession. Our final sample size was comparable with similar qualitative studies [30,36,37].

### Ethical Considerations

Ethical approval for this study was obtained from the Newfoundland and Labrador Health Research Ethics Authority (HREB -2017-194). Before agreeing to participate, all subjects were informed about the nature of the research project, possible risks and benefits, and their rights as research subjects. All participants completed a written consent form. They were also given a copy of the consent form.

### Data Collection

We conducted individual semistructured interviews to gain an understanding of the everyday life-worlds of HCPs in relation to using mHealth [38,39]. Using semistructured interviews allowed the interviewer to begin with a broad question to direct the focus of the interview and then to provide an opportunity for the HCPs to bring forth their thoughts and feelings about the phenomenon that they thought were important [38,39]. The interview prompts are available in Multimedia Appendix 1. If participants identified that they have not used mHealth, they were asked questions pertaining to why they had not used mHealth (barriers). However, we did not ask them about facilitators because they did not have the experience to answer these questions. To facilitate discussions, the interviews were conversational in nature and items were not asked verbatim or in the order presented. As the study progressed, emerging issues were explored with subsequent participants to refine the themes.

The prompts were informed by findings from the literature and input from the authors who have diverse backgrounds including mHealth, pharmacy, nursing, medicine, respirology, family medicine, education, and qualitative research.

The interviews were recorded to enable transparent and accurate transcription. Interview lengths ranged from 20 to 60 min. Topics included the following: demographics, mHealth usage, perceptions toward challenges of mHealth adoption, factors facilitating mHealth adoption, and preferences regarding features of the mHealth intervention for COPD management. Owing to the large amount of data, preferences regarding features of the mHealth intervention will be published in another article. Data consisted of more than 13 hours of interview time with approximately 300 pages of transcription.

### Data Analysis

The interviews were transcribed verbatim and compared against the digital recordings to ensure the accuracy of the content. Identifying information (names) was removed to protect anonymity. We used NVivo (version 12; QSR International) to organize the data and examine the words, including frequency counts, as in classical content analysis [40]. All data were analyzed, but we only coded the data that were relevant for answering the research questions, as recommended by Saldana [41], Wolcott [42], and Yin [43]. An audit trail was created to keep track of all analytic decisions [44].

After using NVivo, we used first cycle coding with the nurses’ data that were both structural and holistic [41], meaning that we used the interview prompts and the literature to guide some of the coding. One researcher analyzed the transcripts and developed a set of themes and subthemes and then obtained input from a second researcher. In the second cycle of coding, the 2 researchers independently coded the nurses’ data using pattern coding to develop themes [41]. They then discussed commonalities and differences in their coding and theme development until a consensus was reached. The analysis of the nurses’ data was mainly inductive and iterative throughout as we went back and forth among the data, the coding, and the themes [45].

After the nursing analysis was finished, we completed the same 2 cycles of analysis for the pharmacist and physician data. These 2 analyses included inductive and deductive analysis. However, the analysis was more deductive in nature as themes had already been developed from the nursing data. The iterative process continued as these analyses were conducted to find commonalities, differences, and new patterns in thinking in relation to the nurses’ data. Once these 3 sets of analysis were complete, the 2 researchers discussed common and different trends among the 3 HCP groups to develop final themes that encompassed all the HCPs.

## Results

### Demographics

The sample included HCPs who worked with patients with COPD in various settings, including respirology clinics, cancer clinics, critical care, long-term care, and community health. Some HCPs founded a medical technology company or had a software programming background. About half of the HCPs had experience with an mHealth intervention to manage COPD. Participant demographics are outlined in Table 1.

The majority of HCPs thought that mHealth can play a role in COPD management; however, some HCPs had opposing views. One nurse who implements an mHealth intervention to manage COPD indicated that “...the majority of our patients are very sad to leave the programme.” However, one physician expressed his concern:

There hasn’t been a lot of evidence to prove that this makes a difference in terms of patient outcomes...I think those people are just happy to have another set of eyes watching them, right. I think it probably gives them reassurance.

Finally, a pharmacist said,

There’s obviously going to be some patients who don’t want to do it who are technology averse in which case that’s totally fine, they can use the traditional methods.

We developed themes under 2 categories: facilitators and barriers that would influence the feasibility and use of mHealth. Table 2 summarizes the main facilitators and Table 3 summarizes the main barriers. We have also included details and examples to illustrate the HCPs’ thoughts and beliefs.

**Table 1:** Participant demographics.

Demographics	Sample size	Age (years), mean (SD)	Years of experience, mean (SD)
Nurses	5	47.3 (6)	19.6 (9)
mHealth^a,b^ nurses	5	40.6 (10)	15.8 (10)
Physicians	5	37 (9)	8.4 (8.7)
mHealth physicians	5	41.2 (12)	14.4 (11)
Pharmacists	7	35.7 (11)	11.4 (10)
mHealth pharmacists	3	27.5 (4)	3.6 (2)

^a^mHealth: mobile health.

^b^Experience in using a mobile health intervention.

**Table 2:** Themes with specific examples regarding the facilitators of mobile health (mHealth) adoption.

Theme	Specific examples for each theme
There are possible health benefits for patients	Patients can become more readily educated about their disease; In areas with limited access to health care, mHealth technologies can bridge the gap between patients and health care providers; Patients can become more motivated, empowered, and accountable with managing their health care
The software needs to be easy to use	The technology needs to be simple; The language should be basic; The software should be visually appealing
Health care providers and patients need to be educated on the use of mHealth	Educational strategies are needed
The credibility of mHealth should be evident	Evidence about the effectiveness of mHealth is important; The credibility of the developer is important
mHealth should reduce the cost to the health care system	It results in a decreased use of health care resources; It is affordable owing to the reduced cost of medical devices, and it does not include a large physical infrastructure; Partnering with private entities could facilitate uptake

**Table 3:** Themes with specific examples regarding the barriers of mobile health (mHealth) adoption.

Theme	Specific examples for each theme
There are technical issues with mHealth	It may include equipment malfunction, password issues, and interoperability; It requires internet access; Many clinics are paper based
There may be privacy and confidentiality concerns	People, other than the patients, might gain access to private information
Lack of awareness is a challenge	Many HCPs^a^ and patients are not aware of the current advancements in mHealth
There may be limited uptake from the elderly	Some HCPs thought older age may be a barrier to technology adoption; Some believed the upcoming generation will be more familiar with technology
mHealth may limit the personal connection between HCPs and patients	Some thought personal connections are necessary; Others thought the advantages of mHealth outweigh personal connections; Others thought a hybrid approach might be optimal
There are possible financial barriers; There were a few challenges mentioned by a minority of HCPs	This includes the high cost of the mHealth intervention, time consumption, and lack of billing codes for HCPs; These included false sense of security, anxiety, lack of motivation, and loss to follow up.

^a^HCP: health care provider.

### Facilitators

#### There are Possible Health Benefits for Patients

Pharmacists, nurses, and physicians agreed that mHealth has the potential to provide health benefits to patients. One nurse, who was experienced with mHealth remarked,

It could make life for them, you know, much easier, and improve their quality of life.

Another nurse felt confident that patients would be “more educated about their diseases and about what things they should be looking for.” A physician commented on his patients who were enrolled in a mHealth intervention program,

...I think those people are just happy to have another set of eyes watching them, right. I think it probably gives them reassurance.

Some HCPs mentioned that mHealth could increase patient autonomy through simulating empowerment and motivation in patients. The following physician statement represents thoughts from several other HCPs, “it would give patients the power to then be a part of their management plan, which is better when patients are empowered, because they feel in control of their health.” A few pharmacists also mentioned increased motivation as part of this same vein of thought and talked about “access to motivation or making the patient really feel like they were more kind of involved in their own healthcare.” Some nurses indicated that mHealth interventions could provide a sense of accountability:

There’s a sense of accountability I believe from the patients. The nurse is watching me this morning, I better do it because she’ll be waiting or he’ll be waiting, definitely.

Access to health care in rural areas was also thought to be an important facilitator. Many HCPs highlighted the importance of mHealth in reducing travel time and improving access to rural areas (Newfoundland and Labrador, Canada). A nurse observed,

You look at all these small communities in and around the island, those people could certainly benefit from some kind of remote monitoring.

This thought was reinforced by others, as in this physician statement,

I think that is probably the best benefit from Mobile Health in this province is that it can reach some of those rural communities where we can’t go and see patients.

As part of rural health care, it was consistently noted that mHealth would make it easier for HCPs to provide care. For example, a nurse pointed out that mHealth would help with management of time and perhaps allow for more patients to be monitored, as the following comment demonstrates,

...what they can achieve in a video appointment is sometimes quicker, and a bit more targeted and efficient, and they can fit them in within their other appointments.

mHealth should reduce cancelled appointments and hospital visits as patients would not have to leave their homes, in urban as well as in rural areas. One physician expressed this concern about hospital visits, coupled with the advantage of mHealth:

...you can just send that from home. Not even have to go into a facility. And sometimes that’s really onerous for people, especially people who are suffering from COPD, so they’re going to have shortness of breath and exertion and find it even harder to get from the parking lot into the hospital, so the more you can do to make their lives easier, it’s great.

#### The Software Needs to Be Easy to Use

Usability was highlighted by the majority of HCPs as an important factor in increasing the uptake of mHealth. One nurse with experience in conducting mHealth interventions cautioned that patients may stop using the intervention owing to usability issues:

...they found it hard, I’d say largely related to the technology, not being able to handle it or finding it too much work. Too tiring, too much trouble, not for them, that kind of thing.

Thus, most HCPs recommended the software to be easy to use. As another nurse pointed out:

...people are overwhelmed when they are diagnosed with something that is new and complicated, and affects something as important as your breathing. So, this has got to be something that is easy for them to access and, I think, easy for them to see benefits from.

Some pharmacists recommended using simple language to enhance usability, as in “it needs to be kept useful, but also simple enough for them to be able to navigate and use.” One nurse reinforced this notion and thought the language should be “set at a grade six reading level, so there’s no issues with comprehension of what they're being asked or told.” It was also thought that the software should be visually appealing, with color and perhaps daily progress or weekly tracking graphs. Font size was also raised as an issue. One nurse quipped, “people my age and above can’t see. A lot of it is very tiny, so the need for reading glasses.” This was apt as COPD generally develops in later stages of life.

In addition, one nurse with experience in mHealth interventions said HCPs may not use the intervention if it was difficult to use, “where the provider is getting all this information, doesn’t feel that comfortable sorting through it, or using it to make clinical decisions, and then it just is going to no use it.” So, users and providers need software that is easy to use as well as comprehensive. To streamline the physician workflow, one physician suggested that data collected by the mHealth intervention should be accessed via the electronic medical record: “I have an electronic medical record so it would be nice if it was actually in electronic format.”

#### Health Care Providers and Patients Need to Be Educated on the Use of Mobile Health

It was recognized that mHealth is a different type of learning for many HCPs as it includes learning about technology instead of diseases. However, as one nurse rationalized, “we need to make sure we are staying up and current and on top of this.” Many strategies were suggested for educating HCPs, such as integrating information about digital health and mHealth in school curricula; self-learning; Web-based learning; learning from coworkers, students, and sales representatives; attending educational sessions; and hiring coordinators for support.

The necessity to educate patients was also acknowledged. As one pharmacist suggested,

I guess most patients with COPD are older and would probably benefit from someone walking through the app with them and showing them how to use it.

Hands-on learning, supplementary print materials, and a video tutorial were suggested as ways to teach patients how to use the software. Others mentioned the convenience of having family support as an enabler.

In terms of who should teach patients, it was thought by some that HCPs should share the responsibility. As advocated by one pharmacist:

I guess anyone, if you're seeing a patient or person who is in need of that service could introduce it. I don’t think one person should have to take all the responsibility, or one profession.

However, this was not an agreed upon idea. Some thought there should be designated people to teach the necessary skills, but there were differing opinions about which group of HCPs should lead the patient education. It was also recommended by some that technical support staff be available as a resource for patients to call when they needed technical help.

#### The Credibility of Mobile Health Should Be Evident

HCPs thought that the credibility of mHealth needs to be made evident to HCPs and patients. This would help raise awareness to facilitate uptake by HCPs. A physician worded it like this,

...if I perceived that this is something that would help someone exercise a little bit more, control their weight, watch their diet, then I would recommend that.

A nurse was even more specific in terms of evidence:

...it would be really important to have some solid, really good evidence to show that, in actual fact, we receive excellent outcomes in terms of quality of life indicators, activity levels, medication usage at a specific time point, be it within one or two years, to decide that this type of monitoring, and this type of connection with your provider is making a difference to your outcomes. I think that type of evidence is what’s going to change my mind as a practitioner about whether it’s worth using it or not.

This sentiment was reiterated by a pharmacist who thought that “knowing if there’s evidence to actually support its use” was essential.

In addition, the credibility of the developer was mentioned, as in this statement from a pharmacist, “it’s also about the credibility of who’s putting the app together.” Added to this, recommendations from credible HCPs were also thought to be important. One physician commented:

I mean, the power of one's network. If I view something and I think that it’s good, then me giving it a vote of confidence that would then get shared, and people would know that I am independently choosing to recommend something.

#### Mobile Health Should Reduce the Cost to the Health Care System

It was thought that mHealth has the ability to provide the “clinical assessment and healthcare that was required in a more cost-effective manner”, as recommended by one of the nurses. It should decrease emergency visits and hospital admissions, as explained by a nurse who thought it would “hopefully catch things in the earlier stage before these patients who were mostly elderly got in enough trouble that they would end up in the emergency department.”

Advancements in mHealth can result in a decrease in expenses, as a third nurse explained,

I can send a patient a whole set of devices including a blood pressure cuff, O2 sat machine and a weigh scale for less than 300 dollars.

Large physical infrastructure would not be required, and it was suggested that some of this could be outsourced to private entities that are already doing this type of work, thereby reducing expenses to taxpayers.

### Barriers

#### There Are Technical Issues With Mobile Health

Many HCPs expressed that technical issues can be barriers for mHealth adoption. Specifically, equipment malfunction, password issues, and interoperability were mentioned frequently. For example, a nurse reported,

There’s been issues with the technology not communicating because we have setups in four different ways.

In addition, some technical specifications are required, such as the smartphone being Bluetooth compatible, along with cellular and Wi-Fi connections being available. One nurse elaborated,

...there are patients within little pockets of...that don't have cellular service or Internet connection, so unfortunately those patients will not be able to be referred to the program.

Another limiting condition to sharing mHealth data via electronic medical records was mentioned by physicians, in that many clinics are still paper based or not up to date in technology use.

#### There May Be Privacy and Confidentiality Concerns

A few HCPs thought privacy and confidentiality could be a barrier to mHealth adoption. A pharmacist, echoing other HCPs, questioned,

How are patients confident that the information that’s in that app is only going to stay with them and that other people are not going to see that data?

The concern of family members viewing private information was raised,

Patients, you know, if they’re competent they don’t want their family members to see their information and that could be an issue.

Also, the issue of stolen or lost phones that contained private information was raised. However, other HCPs thought these issues could be mitigated with security, as expressed by a pharmacist,

...if it is secure and the patient gets to decide who accesses it, I don’t see it being an issue with confidentiality.

And, some HCPs, as noted by a nurse, were ambivalent regarding privacy and confidentiality,

I wouldn’t imagine that there are any more privacy concerns than there are with anything else within health care.

#### Lack of Awareness Is a Challenge

Many HCPs indicated that lack of awareness is a major barrier to mHealth adoption. One physician with limited mHealth experience expressed this concern,

I think if that had been a part of my training more and I’d seen it more then it could definitely become part of my own training.

Employers’ lack of knowledge was also mentioned. For example, a nurse shared:

Our employer doesn’t want to see us having them out, people will have the impression we are using it for personal use. That is one big factor. Our employer tells us, keep your phones hidden, don’t have them out.

#### There May Be Limited Uptake From the Elderly

HCPs had conflicting opinions regarding age and mHealth adoption. All physicians and some nurses and pharmacists agreed that the elderly may face issues in adopting these technologies, as indicated by this pharmacist,

A lot of the patients with COPD being older and maybe not as app-savvy as the group that you’re aiming towards.

This thought was reinforced by one physician’s words, with a caveat of doubt,

I suppose I would assume that the elderly and the more frail would not be tech-savvy, though, I know smartphone use is increasing with the ageing population.

This caveat was supported by some of the nurses with an mHealth experience, as expressed by one experienced nurse:

I had patients who are older than 90 who never owned a computer in their life and managed to do their sessions on their iPads and send it to me with no trouble. So, I think it depends on maybe education level and understanding, and maybe how things are explained to them.

A couple of pharmacists experienced in mHealth even stated that some elderly people have embraced technology:

I’ve had a lot of kind of older generation patients that once we’ve kind of sat down they’ve said oh I’ve been tracking this or I have this app, and I was kind of shocked. So until you kind of try it out and recommend it to people you never know what they’re open to using or what they’re already using.

It was also thought by some that the upcoming generation will be more familiar with technology, as a nurse surmised,

We have to be sensitive to the fact that technology is present in my world, it’s present in yours, but it wasn’t in my grandparents.

Some physicians also thought that future generations will value and use mHealth more than the current generation,

I think the younger generation will, you know, take this in very easily and very much accept it, so I think going forward there’s only going to be more of it, not less.

It was also posited that some older HCPs may face issues when adopting mHealth, as put forward by one pharmacist:

I’m sure there’d be some potentially older pharmacists who are less familiar with smartphones and apps that might have more trouble, and may benefit from a tutorial type thing.

This was reiterated by a physician:

I think that probably technology maybe gets pushed to the side. I think that a lot of the physicians too might be, not scared but reluctant to use technology and to learn a new skill, especially if they’ve been in practice for thirty years or something.

#### Mobile Health May Limit the Personal Connection Between Health Care Providers and Patients

As with age, HCPs had conflicting opinions about mHealth and building personal connections between patients and HCPs. A nurse who worried that mHealth might limit the personal connections said:

I like to have a bit of actual contact and eye contact, and hear the tone of someone’s voice, and a gentle touch sometimes can be so reassuring, you know. I think it’s going to be lost with this type of technology.

However, this same nurse added that even minimal contact could mitigate that barrier, as in “I think there needs to be some sort of human contact, even if it is just the face of the person who receives that information.”

Some physicians also agreed that mHealth lacks this type of contact, as in “I don’t think you’re ever going to really replace that human element.” However, although it was emphasized that interacting with patients face to face is better than online, some HCPs struggled with the advantages of human contact versus access. One nurse who was a champion of human contact, recognized that mHealth is:

...increasing access and to me, that would be a better benefit than the actual face to face, to be able to reach more people more often.

Then there were nurses experienced in mHealth who thought it could enhance the personal connection, as in,

I think the bond is actually a bit more in this program than it was when I was a bedside nurse in some ways, because you're getting more personal with the patient about other aspects of their healthcare as well.

One nurse reported that she had done surveys about patient satisfaction, provider satisfaction, and support staff satisfaction and “the surveys, they do come back that it’s similar if not better than a face-to-face.”

The majority of physicians, and many pharmacists, thought mHealth has the potential to improve personal connections. This pharmacist’s statement represents a commonly expressed example of how this could happen:

I think it would strengthen that relationship because you could ask them about their apps and go through it with them when they come other than just seeing if they’re late using their prescriptions or late picking them up or anything.

Some HCPs suggested a hybrid model so that mHealth could supplement the personal connection, as in this physician’s comment,

Do I think that it could totally replace it, absolutely not...but can I see it being hand-in-and, absolutely.

This sentiment was supported by another physician:

I would like to see them for the initial consultation, but I think for follow-up reports, you know, we could save them great distances from travelling.

This was also supported by a nurse:

I think having regular face-to-face contacts intermittently is still a very important part of healthcare, and it’s something that I think will never be completely removed.

#### There Are Possible Financial Barriers

HCPs had conflicting opinions about financial implications. A few HCPs said some patients with COPD may not be able to afford mHealth and do not have access to smartphones. One physician expressed it this way,

Generally more patients with COPD are falling in the lower socio-economic grouping that wouldn’t necessarily be able to afford this.

A nurse with experience conducting mHealth programs endorsed this concern by adding that only about 10% of participants may remain in the mHealth intervention if the insurance company stopped paying for the service.

In addition to individual patient costs, there is the initial cost of establishing the infrastructure, including costs related to storing data in the cloud. In addition, costs related to the maintenance and replacement of outdated technology were discussed, as reinforced by one nurse,

There’s a number of equipment across the province that are nine years old, and if they die then there’s no replacement.

In addition, some mHealth programs are limited to a certain period. Participants may get medical devices (eg, blood pressure monitors and pulse oximeters) that have to be returned for cleaning to be used by other participants. One experienced nurse complained that getting medical devices back from patients can be problematic, as in:

I have actually been at the plants, the facilities where we get them back and clean them. Andcockroachesin the boxes that were coming back and just swilled with feces and blood and so on. It is just... They have been horrendous.

Most physicians thought lack of time was a major challenge. They mentioned time to learn about mHealth themselves, time to teach patients, and time to review the results of the mHealth intervention. One physician gave this example:

When you get a 12 page report on one patient and you’re seeing 40 patients a day and you know time constraints with the amount of work that you do outside in terms of paperwork is already a burden.

Pharmacists had contradictory views about time. A few pharmacists thought lack of time could be a barrier, as in:

Most pharmacists are quite busy as it is... I see the workload potentially going up because now if patients are using this they can’t forget to write things down or lose what they documented. It’s all there for them, so now they bring the information in.

Alternatively, other pharmacists thought that mHealth could save time by collecting information required in advance “with the expanded pharmacists role we’re building more time to spend with our patients and in that sense we will have that time to teach them and to monitor some of these new technologies that are coming up.”

Lack of reimbursement and billing codes were also mentioned as barriers. One experienced pharmacist explained:

...there’s not a whole lot of reimbursement for services like this and like that’s the biggest barrier with most things within the pharmacy profession...doing like daily monitoring on patients like is time-consuming and we definitely want to do it but unfortunately like it does take time and resources and those resources aren’t always available.

In addition, it was emphasized that the lack of billing codes for mHealth is another financial barrier, as a physician insisted:

I mean we’re all so busy that nobody wants to do anything for free because why would I do that for free if I get paid for it. So that’s a barrier that has to be overcome is that how do you change some of the way physicians are paid. There’s no incentivizing the optimized care as an example. If I do a poor quality of care for my COPD patient or if I do an excellent quality of care, it’s the same payment. So there’s a problem with the system in that sense and physicians in general would be resistant to sort of evaluate how well they’re doing with their patients.

#### There Were a Few Challenges Mentioned by a Minority of Health Care Providers

There were additional challenges that were mentioned by small numbers of HCPs. For example, one physician thought patients may gain “a false sense of security” about their health status, owing to technology. Another physician voiced concern that,

...some sub-groups of patients with anxiety might have impaired quality of life because then they become obsessed with that rather than actually just saying okay that’s what they’re saying, I’m okay.

A pharmacist questioned validity:

...the validity of the data would be something that some people might question. I guess a lot of that would depend on how straightforward the devices are to use or how much training might be required to make sure that they are using it correctly.

Motivation to continue using the intervention was also a concern. A pharmacist wondered,

I think getting patients to use it and use it often enough might be difficult, depending on the patient.

A few pharmacists and physicians noted that many patients with COPD are not motivated to manage their disease. One pharmacist commented:

The biggest challenge I find with COPD patients, now that’s the population that I deal with, is that they are smokers and continue to smoke, the majority of them. Their education level is probably a little bit on the lower side and that’s related to the whole smoking, right, that kind of thing, the socio-economic status of the patient. So they’re not necessarily invested in improving their health with a lot of effort, right. They’ll take an inhaler, take a pill to help them get better, but really changing their lifestyle and their smoking is not high on their list.

One nurse highlighted that about 30% of patients dropped out after using an mHealth intervention.

## Discussion

### Principal Findings

This qualitative study found that HCPs, in general, had a positive attitude toward mHealth adoption for COPD management, but several facilitators and barriers were identified. More barriers were identified than facilitators, indicating a need to address these barriers to optimize successful implementation of mHealth interventions.

To facilitate mHealth uptake, our thoughts, based on the data, are that both HCPs and patients need to understand the potential benefits of the mHealth intervention. The interventions must be easy to use for both patients and HCPs. This could reduce the time and resources required to teach patients and providers about the mHealth intervention. One physician stated that the use of mHealth interventions could provide a false sense of security, thereby keeping the user from seeking medical advice in a timely manner. This concern along with the lack of awareness concerned HCPs, an important finding is the need for HCPs to teach patients about mHealth interventions. Some HCPs thought there should be a designated person to teach patients. It is preferable that these professionals have a background in chronic disease management and technical support.

There were a few barriers identified by the HCPs. Most of these barriers have the potential to be resolved, as suggested by many of the HCPs. Technical issues continue to be a challenge for mHealth adoption, especially for rural areas and developing countries that have poor connection network.

### Comparison With Previous Work

Although the numbers of HCPs using mHealth interventions are growing, studies focusing solely on the frontline staff perspective on mHealth are limited [36,46]. Some of the findings presented in this study confirm findings that have been reported previously in the context of mHealth for COPD management. As Damhus et al [36] noted, HCPs reported technical issues as a major challenge for mHealth adoption. Our findings are in agreement with Vorrink et al [47], who stress the importance of training patients and HCPs on the proper use of mHealth. In this study, as well as other studies, we have noted that mHealth will not replace face-to-face interactions [30,36,47]. In agreement with Damhus et al [36] and Korpershoek et al [30], we suggest that the expected benefits of using mHealth contribute to the success of mHealth uptake, although our study provides additional insight with regard to these perceptions.

### Strengths and Limitations

There are several strengths of this study. First, this research is based on a diverse sample of participants. It includes various perspectives by presenting the views of nurses, pharmacist, and physicians, including a respirologist. This human-centered approach ensures that needs and challenges of different people involved in the management of COPD can be considered before developing an mHealth intervention. Second, some HCPs had experience in using an mHealth intervention to manage COPD which further increases the richness of the data. Third, all of the interviews were conducted in a similar manner to ensure consistency during the data collection and analysis. Finally, mHealth is particularly important in geographic locations with relatively large proportions of rural residents such as Newfoundland and Labrador. mHealth may enhance care provider access throughout sparsely populated rural areas. Newfoundland and Labrador has a substantial remote and rural population, therefore our results may be more applicable to rural areas.

There were also several limitations. First, not all the HCPs had experience with using mHealth. Thus, the perceptions of these participants were not based on actual interventions with patients. Second, we used only one data collection method, thus not triangulating data collection. Conducting focus groups with some of the participants following the individual interviews could have yielded richer information as participants would have been given the opportunity to compare their thoughts and confirm or expand upon each other’s ideas. This would be a recommendation for a future study.

### Implications for Practice

The findings of this study provide insights into the barriers and facilitators for using mHealth as a part of COPD management. This information may help a variety of stakeholders who are planning to use mHealth interventions for COPD management. Lessons learned include the importance of raising an awareness among patients with COPD and HCPs regarding the potential of mHealth interventions in COPD management. Professional associations and universities could play a significant role in raising an awareness of, and even introducing, mHealth in undergraduate health professional curricula. It also may be beneficial to designate an HCP, with a background in chronic disease management and technical support, to teach patients about mHealth.

The findings emphasize the importance of developing a user-friendly mHealth intervention. This could reduce the time and resources required to teach patients and providers about the mHealth intervention. In addition, the lack of an internet connection limits access to mHealth interventions, so this should be taken into consideration when measuring access to health resources in rural communities.

In terms of credibility, health organizations such as the Food and Drug Administration, Health Canada, or the Canadian Association for Drugs and Technologies should take an active role in regulating mHealth interventions. These organizations can develop their own app stores, similar to the Veteran Affairs app store, to showcase credible mHealth interventions. In addition, when developing mHealth interventions, it is important to follow international guidelines for the exchange, integration, sharing, and retrieval of electronic health information [48]. This could help in addressing interoperability issues. Nevertheless, these regulations should be implemented in a manner that supports mHealth uptake.

### Recommendations for Future Research

Future studies would benefit from conducting focus groups with some of the participants following the individual interviews. Focus groups could yield richer information as participants would be given the opportunity to compare their thoughts and confirm or expand upon each other’s ideas. Furthermore, including the perspectives of allied HCPs, such as physiotherapists, social workers, and occupational therapists, would be beneficial to understand the perspectives of administrators (eg, information technology managers) who may be able to identify some of the challenges with using mHealth for COPD management. The authors have conducted a similar study with a focus on the perspectives of individuals with COPD. In addition, a future article will focus solely on the features of the ideal mHealth intervention for COPD management. After developing a user-centered mHealth intervention, the authors recommend using a mixed methods framework for usability testing [49].

### Conclusions

It is important to understand the perceptions of HCPs regarding the adoption of innovative mHealth interventions for COPD management. This study identifies the facilitators and barriers that may aid in the successful development and implementation of mHealth interventions for COPD management. Lessons from this study may also be applied to other chronic diseases. Additional research is needed to investigate the conflicting opinions regarding mHealth adoption by the elderly, the personal communication between HCPs and patients, and the cost-effectiveness of mHealth interventions in COPD management.

## Appendix

Multimedia Appendix 1 Health care provider interview prompts.

## Abbreviations

COPD: chronic obstructive pulmonary disease
HCD: human-centered design
HCP: health care provider
ISO: International Organization for Standardization
mHealth: mobile health
PI: primary investigator
