# Supplementary material for: Perceptions of Health Care Providers Regarding a Mobile Health Intervention to Manage Chronic Obstructive Pulmonary Disease: Qualitative Study
Source: JMIR Mhealth Uhealth. 2019 Jun 10;7(6):e13950. doi: 10.2196/13950 (PMC6592475; doi:10.2196/13950)
Supplement: Multimedia Appendix 1 [file mhealth_v7i6e13950_app1.docx]

## Appendix 1- Healthcare Provider Interview prompts

- Date and place
- Age and experience from general practice
- Job title

**General perceptions**

- Tell me a little bit about your experience with patients with COPD.
- What are the likely treatment outcomes?
- Do you know what mHealth is?

**Facilitators**

- Have you used mhealth before? (if no, go to barriers).
- Tell me about a situation when you have tried to use mhealth in your practice.
  - Why this episode?
  - What did you use it for?
  - What data did you collect?
  - How often?
- What motivated you as a healthcare provider to use mhealth?
- What elements of the intervention do you think are most important?
- Did you do any preparation before using mHealth to manage COPD?
- What is necessary for you to obtain knowledge/experience and keep up to date about mHealth?

**Barriers**

- What is limiting you from using mHealth in your practice?
- What difficulties have you experienced when using mHealth?
- How did you solve it?
- Were there any challenges?
  - (financial, employees, technical)?
- Did you experience changes in the contact/bond with the patient?
- Do HCPs require education before use and how did that happen in the past?
- Do patients require education before use and how did that happen in the past?

**mHealth in COPD Management**

- Do you see a role of mHealth in COPD management?
  - how do you feel about apps used in COPD management?
- Could you tell me about whether you would be interested in using it?
- How do you perceive using mHealth to manage COPD?
- What information do you want to collect from the patient?
  - Education (how often)
  - Survey (how often)
  - Care plan
  - Compatible medical devices (e.g. spirometer, pulse oximeter, medication adherence device)
- What else would you like it to do?
- What would you change, take away or add?
- What about any problems or concerns you can see with this?
- How does mHealth affect the current COPD management process?
- Did you have to do any practical changes in consultations to enable the intervention? (Time, follow-ups, other?)
- How about viewing large amount of data, e.g. heart rate, spirometry, survey?
- Does your patients see a role of of mHealth in COPD management?
- How does your patients perceive using mHealth to manage COPD?
- Who do you think can be a candidate for this intervention? (What is it about the patient that makes them suitable or not?)

**Final questions**

- Would you recommend mhealth to a colleague?
  - if so, what would you emphasize?
- Would you like to add anything?
- Would you like to elaborate on anything I asked?

Thank you for participating in this study, your answers to these questions are very important to us , and we really appreciate you taking the time to complete this interview. Please contact me if you have any questions or would like to discuss this topic further.
